# Increased accuracy of FNA-based cytological diagnosis of pancreatic lesions by use of an ethanol-based fixative system: A STROBE compliant study

**DOI:** 10.1097/MD.0000000000030449

**Published:** 2022-09-09

**Authors:** Martin Bürger, Antje Heidrich, Iver Petersen, Andreas Stallmach, Carsten Schmidt

**Affiliations:** a Clinic for Internal Medicine IV (Gastroenterology, Hepatology and Infectious Diseases), Jena University Hospital, Jena, Germany; b Clinic for Gastroenterology and Hepatology, Faculty of Medicine and University Hospital Cologne, University of Cologne, Cologne, Germany; c Dr. med. Kielstein, Ambulante Medizinische Versorgung GmbH, Jena, Germany; d Institute of Pathology, Jena University Hospital, Jena, Germany; e Institute of Pathology, Waldklinikum Gera, Gera, Germany; f Medical Clinic II (Gastroenterology, Hepatology, Endocrinology, Diabetology and Infectious Diseases), Fulda Hospital, Fulda, Germany; g Medical Faculty of the Friedrich Schiller University, 07747 Jena, Germany.

**Keywords:** endoscopic ultrasound, fine needle aspiration, liquid based cytology, pancreatic cancer

## Abstract

EUS-guided fine needle aspiration cytology (FNA) is the gold standard of evaluation of solid pancreatic lesions. However, accuracy is generally low. The aim of this study was to compare the diagnostic yield of conventional cytology (CC) with liquid-based cytological analysis using an ethanol based fixative system (LBC) without onsite cytopathological assessment. We performed a retrospective evaluation in patients referred to the Department of Interdisciplinary Endoscopy at Jena University Hospital for FNA of pancreatic masses between 2008 and 2015. LBC preservation of specimen was introduced in April 2011. Gold standard was defined as a surgically obtained histology or a patient follow-up of at least 1 year for diagnosis or exclusion of malignancy. 172 patients were included into the final analysis. Mean age was 64.8 years (SD 12.4 years), 105 patients were male. 107 lesions were malignant, while 65 lesions were benign. 89 specimens were evaluated by CC, whereas 83 specimens were processed by LBC. Liquid-based cytology performed significantly better than conventional cytology in terms of sensitivity (87.8% vs 67.2% (*P* = .021)), specificity (100% vs 87.1% (*P* = .047)) negative predictive value (NPV) (85% vs 58.7% (*P* = .009)) and accuracy (92.8% vs 74.2% (*P* = .001)). We observed no learning curve after implementation of LBC Liquid based cytology is a simple and inexpensive technique that helps improving sensitivity, specificity, NPV and accuracy over conventional cytology in fine needle aspirates from patients with pancreatic lesions. Therefore, this real-world evidence shows, that EUS-FNA specimen processing should be performed using LBC to achieve best possible results.

## 1. Introduction

Despite of advances in imaging modalities and improving techniques concerning tissue characterization, pancreatic masses with possible underlying malignancy remain a diagnostic challenge. In the case of a pancreatic ductal adenocarcinoma (PDAC), accounting for more than 90% of pancreatic cancer cases, an average 5-year survival rate of <10%^[1]^ is to be expected. Surgical resection is the only curative treatment option, however, morbidity still occurs in up to 40% of patients undergoing open pancreaticoduodenectomy.^[2]^ To preserve curative treatment options, to initiate neoadjuvant or even palliative therapy in case of malignancy or to prevent unnecessary surgery with potentially high morbidity and even mortality, it is of major importance to achieve an accurate diagnosis from endoscopic tissue sampling. EUS-guided fine needle aspiration (EUS-FNA) is a widely available technique and demonstrated to be safe and useful for tissue sampling of unknown solid and cystic pancreatic masses.^[3]^ Even though EUS-FNA is recognized as gold standard for pancreatic tissue acquisition, cytological assessment of specimen faces various problems. For example, chronic pancreatitis may cause atypical histological alterations making a distinction from well-differentiated neoplasia impossible. Moreover, in case of pancreatic masses such as autoimmune pancreatitis cytological specimen do not allow adequate evaluation.^[4]^ In addition, cytological assessment itself is a challenge for the cytopathologist, in particular for less experienced investigators in the field, while dedicated pathologists achieved a higher level of diagnostic accuracy.^[5]^ Different techniques concerning FNA tissue acquisition have been evaluated in recent years to improve its diagnostic yield, for example techniques for targeting lesions (e.g. fanning or torque technique), different sizes of needles, needle characteristics, number of passes, suction technique, etc.^[6]^ It has also been shown, that a cytopathologist evaluating recently obtained specimen directly in the endoscopic department (rapid on-site examination (ROSE)) increases the diagnostic yield,^[7]^ but in most european countries, this approach is not available in daily clinical care. In addition, handling of the obtained samples has the potential to improve (or deteriorate) diagnostic accuracy. Current standard of diagnosis is a direct smear of the obtained specimen on glass object plates prepared by the endoscopist that is directly send to the evaluating pathologist.

The aim of our study was to compare the diagnostic yield of an ethanol-based fixative system including a cell block procedure^[8]^ compared to conventional cytology in a large cohort of patients with pancreatic lesions. Furthermore, we aimed at investigating a possible learning curve while integrating the new approach into daily clinical practice.

## 2. Materials and Methods

### 2.1. Patients

Between January 2008 and April 2015, a total of 191 patients presented to the Interdisciplinary Endoscopy at Jena University Hospital for EUS-guided FNA for further diagnostic workup of solid pancreatic lesions with or without cystic components. There were no inclusion or exclusion criteria, except for feasibility and acceptability of EUS-FNA and an age of at least 18 years.

### 2.2. Endoscopic intervention and techniques

The endoscopic procedures were performed under conscious sedation by faculties and attendants of Jena University Hospital, Clinic of Internal Medicine IV, who were highly experienced in EUS-FNA. Endoscopic ultrasound was performed using a linear array echoendoscope (GF UCT 180, Olympus, Tokyo, Japan). Tissue acquisition was performed by use of a 22G FNA needle (Expect 22G, Boston Scientific, Natick, MA, USA) under endoscopic ultrasonographic guidance. For lesions of the pancreatic head or uncinate process a transduodenal access was chosen, while transgastric access was chosen for masses of the pancreatic body or tail. The needle was inserted into the lesion, then the stylet was removed. Aspiration cytology was obtained with a 10 cm³ suction syringe applied to the hub of the FNA device. The number of needle passes was performed at the investigator’s discretion, usually 4 to 6 passes were carried out. At the end of the procedure the suction was stopped and the needle was retracted. After samples had been visually assessed for adequacy by the endoscopist, samples were prepared for cytological examination.

### 2.3. Final diagnosis and cytological examination

Gold standard was defined as a surgically obtained histology (n = 61) or a patient follow-up of at least 1 year to diagnose or exclude malignancy (n = 111). Pancreatic tumors were considered benign if at least 12 months of follow-up showed no signs of malignancy.

Evaluation of cytological samples was performed by experienced pathology faculty from the Institute of Pathology, University Clinic Jena, Germany, without knowledge of the patient’s history, laboratory results or prior imaging procedures. There was no ROSE performed by an onsite cytopathologist. From February 2008, direct smear of specimen was performed on glass object plates by the endoscopist and directly send to the pathologist for further processing and diagnostics. From April 2011 methods were converted to liquid-based cytology preservation of cytology specimen including a cell block procedure to allow a histology-like processing of the cytological specimen.^[8]^ Liquid-based cytology is an ethanol based fixative system that preserves cells and small tissue fragments in suspension, lyses red blood cells, and allows to perform immunohistochemical staining of cytospin pellets. Therefore, the entire sample was transferred from the FNA needle into 5ml the ethanol-based fixative (BD CytoRich^®^ Red Preservative (Becton, Dickinson and Company, New Jersey, USA)). The FNA needle was additionally flushed with 2 ml of CytoRich^®^ and the sample was then sent to the pathologist for further diagnostics.

Cytological grading of specimen by experienced pathologists in 5 different categories was as follows: category 0: nondiagnostic, insufficient; category I: benign; category II: atypical, favor benign; category III: atypical, suspect malignant; category IV: high grade dysplasia; category V: malignancy.

Results of cytology specimen were then divided into 2 groups, group A: category I + II and group B: category III + IV + V. Group A was considered benign, while group B was regarded as malignant.

### 2.4. Statistical analysis

Results were described as median and range or mean and standard deviation, as appropriate. Associations of parametric continuous data were evaluated using the t-test or the Wilcoxon rank sum test (for nonparametric data). Categorical data were summarized as the percentage of the group total. Fisher exact test (two-sided) or the chi-squared test were used to explore associations of categorical data between 2 groups. A *P*-value of < 0.05 was considered statistically significant. Results were calculated using the IBM SPSSwin^®^ Statistics software, version 24 (Somers, NY, USA).

### 2.5. Ethical statement

The study protocol conforms to the ethical guidelines of the 1975 Declaration of Helsinki as reflected in a prior approval by the institution’s human research committee. In accordance with German law, a written informed consent from the participants due to the strictly retrospective and anonymized design of our study (paragraph 27, sentence 2, Thuringian Hospital Act (ThürKHG) in the version of the notice of 15.06.2018) was not required.

## 3. Results

### 3.1. Patient characteristics

**Figure 1.**
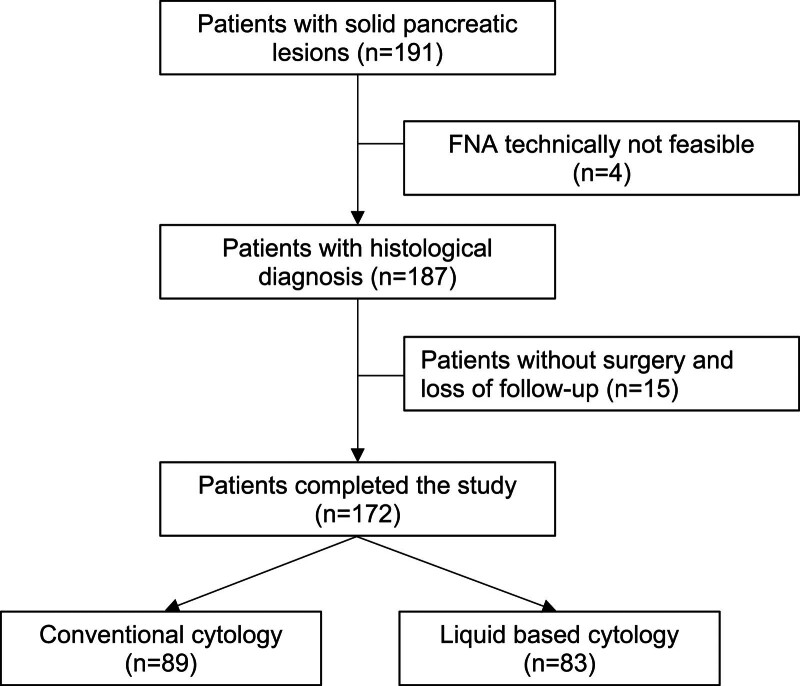
Flowsheet of patients.

191 patients presented for further diagnostic work-up of solid pancreatic lesions. In ten patients repeated FNA was performed with only the first biopsy result taken into consideration. In 4 patients FNA was technically not feasible, mostly due to overlying blood vessels. A total of 187 FNA specimen were successfully obtained. 15 of the remaining 187 patients did not undergo surgical evaluation and got lost to follow up, therefore, 172 patients finally could be evaluated (see figure 1). Patients were between 22 and 93 years of age (mean age was 64.8 years (SD 12.4 years), men represented 61.1% of the study population (n = 105)). 51 patients (29.7%) suffered from diabetes mellitus while in 30 patients (17.4%) chronic pancreatitis had been diagnosed. Distribution of these diseases was not different between diagnostic groups (table 1).

**Table 1:** Patient characteristics and parameters of pancreatic masses.

Patient and tumor characteristics	Conventional cytology (CC; n = 89)	Ethanol-based fixation (LBC; n = 83)	*P*
Age [years (SD)]	63.9 (11.4)	65.7 (13.5)	0.153
Gender (male)	58 (65.2%)	47 (56.6%)	0.276
Comorbidities			
none	52	50	0.184
chronic pancreatitis	6	13	
diabetes mellitus	24	16	
chronic pancreatitis and diabetes mellitus	7	4	
Localization			
pancreatic head	57	54	0.482
pancreatic body	28	22	
pancreatic tail	4	7	
Max. diameter of masses [mm]	35.3 (15.8)	31.1 (13.1)	0.129
Cystic component of masses	19 (21.3%)	32 (38.6%)	0.019
Malignant vs benigne disease [n (%)]	57 (64.1%)	50 (60.2%)	0.639
Distribution of malignant diseases [n (%)]			
Ductal Adenocarcinoma	47	40	0.233
Neuroendocrine tumor	4	8	
Cholangiocellular carcinoma	3	0	
Ampullary cancer	1	0	
Pancreatic metastases	2	2	
Distribution of benign diseases [n (%)]			
Chronic pancreatitis	20	23	0.025
Serous cystadenoma	3	2	
IPMN	0	5	
Mucinous cystadenoma	2	0	
Autoimmune pancreatitis	0	2	
Normal pancreatic tissue	2	0	
Tuberculosis	1	0	
Necrotic tissue	1	0	
FNA cytology unclear	3	1	

### 3.2. Characteristics of pancreatic lesions

Localization of pancreatic masses were pancreatic head and uncinate process (n = 111; 64.5%), pancreatic body (n = 50; 29.1%), and pancreatic tail (n = 11; 6.4%). Mean maximum size of masses was 33.5 mm (SD 15.3 mm). These parameters were not significantly different between specimen evaluated by conventional cytology vs liquid-based cytology. 51 masses (29.7%) showed a cystic structure as part of the lesion, with a higher proportion in specimen evaluated by liquid-based cytology (*P* = .019) (table 1).

### 3.3. Cytological results and final diagnoses

Classification of specimen was as follows: category 0: 0 patients; category I-II: 80 patients, category III: 20 patients, category IV: 23 patients, category V: 49 patients. According to the gold standard 107 lesions were malignant, while 65 lesions were benign. These characteristics were not differently distributed between diagnostic groups (*P* = .639). However, distribution of different benign conditions was statistically different between the 2 groups, while distribution of malignant diseases was not different. Detailed patient and tumor characteristics are summarized in table 1.

### 3.4. Comparison between liquid-based cytology and conventional cytology

Liquid-based cytology preservation method in comparison to conventional cytology led to a significant improvement in sensitivity (87.8% vs 67.2%), specificity (100% vs 87.1%) negative predictive value (85% vs 58.7%) and accuracy (92,8% vs 74.2%), while positive predictive value also showed a tendency towards improvement (100% vs 90.7%; *P* = 0,1162). Data are summarized in table 2.

**Table 2:** Test parameters comparing liquid-based cytology (LBC) and conventional cytology (CC).

	CC	LBC	P
Sensitivity	67.2%	87.8%	0.0207
Specificity	87.1%	100%	0.0465
PPV	90.7%	100%	0.1162
NPV	58.7%	85%	0.0091
Accuracy	74.2%	92.8%	0.0207

### 3.5. Diagnostic yield within 2 time periods after introduction of the new methodology

In order to evaluate a potential learning curve of the pathologist LBC samples were subdivided into 2 groups of about the same size (41 and 42 samples, respectively). In summary, we did not find statistically significant differences with regard to the test parameters. For details, please see table 3.

**Table 3:** Test parameters comparing liquid-based cytology (LBC) during the first and second half of patients after introduction of the new processing technique.

	LBC 1. half	LBC 2. half	*P*
Sensitivity	84.6%	91.3%	0.6707
Specificity	100%	100%	1
PPV	100%	100%	1
NPV	78.9%	90.5%	0.3976
Accuracy	90.2%	95.2%	0.4326

## 4. Discussion

Accurate classification of benign vs malignant etiology of unknown pancreatic lesions is of crucial importance regarding further patient management. In the evaluation of pancreatic masses, EUS-FNA plays a central role obtaining cytological specimens to confirm the diagnosis and has become the gold standard in this regard. Even though universally available and inexpensive it is still infrequently used in EUS-FNA specimen processing. Several meta-analyses showed sensitivities for solid^[9–12]^ and cystic^[13,14]^ pancreatic masses of 85% - 92% and 51% - 65%, resp., and specifities of 96%–98% and 91%–94%, resp.. Even though these performances are promising and the technique is widely available, the reported data largely depend on the endosonographers experience,^[15]^ the experience and volume of the center, presence or absence of an on-site pathologist,^[16]^ concomitant diseases such as chronic pancreatitis, interpretation of indeterminate cytological gradings,^[17]^ number of passes and the needles^[18]^ used.

In our study, we retrospectively investigated the diagnostic yield of EUS-FNA cytology in a large consecutive patient cohort with solid pancreatic lesions of unknown etiology according to the processing of cytological specimen, comparing an ethanol-based liquid fixative system to conventional preparation using direct smear of specimen on glass object plates.

In our study, we achieved a sensitivity of 67,2% using the conventional preparation on glass object plates with a specifity of 87,1%. By use of liquid-based cytology, we were able to increase the sensitivity and specifity significantly to 87,8% and 100%, resp.. Also, this methodical change led to a significant improvement in NPV (85% vs 58.7%) and accuracy (92.8% vs 74.2%), while PPV showed a tendency towards improvement (100% vs 90.7%; *P* = 0,1162). These substantially improved results may result from the fixation solution due to its properties: it preserves cells and small tissue fragments in suspension, lyses potentially disturbing red blood cells and therefore may enable the pathologist to classify cells more accurately through enhanced visualization of cells originating from the pancreatic tumor. Also, specimen handling becomes way easier for the endoscopist who is mostly untrained in specimen processing, since the susceptible production of glass object plates no longer has to be carried out; instead, all of the obtained specimen from the FNA needle is transferred into 5ml ethanol-based fixative by simply flushing the needle, while all further processing is taken over by the pathologist trained in this matter. To evaluate a possible learning curve for the pathologist, we compared the results of the first and second half of the new processing technique but couldn’t find any statistically significant differences regarding the test parameters concluding it is a method that can be easily implemented in daily clinical care.

The pathological assignment of category III to either a benign or malignant classification is still a matter of debate: since histopathological interpretations of suspicious category III specimen differ from 1 study to another, a careful comparison of the diagnostic accuracy of diverse reports is advisable.^[19]^ From a clinical point of view, an assignment to the benign or malignant category is obligatory for further decision-making. In a retrospective analysis, Layfield and coworkers tried to categorize the indeterminate categories “atypical” and “suspicious for malignancy” and actually found malignancy in 79,2% and 96,3%, resp., stating classification of “suspicious for malignancy” as malignant optimizes diagnostic sensitivity and specifity.^[20]^ In our study, 16 specimens were categorized as category III and we therefore interpreted those specimen as malignant. Ex post our clinical approach to interpret category III specimen as malignant has been confirmed by our study results, as 14 out 16 (87.5%) of category III specimen were malignant according to the gold standard.

When comparing different studies concerning the diagnostic performance of FNA, close attention is advisable, since cytological classifications vary between studies and therefore have impact on the results. In their meta-analysis assessing the performance of FNA for diagnosis of solid pancreatic neoplasms, Hewitt et al precisely differentiated the results of the included studies depending on the cytological classification made: they achieved using the same classification as ours a comparable pooled sensitivity and specifity of 91% and 94%.^[21]^ But, this data were achieved by involving rapid onsite evaluation (ROSE), which has a significant impact on diagnostic yield,^[22,23]^ whereas we used LBC without an onsite cytopathologist.

Since new fine needle biopsy (FNB) needles were introduced allowing to obtain core histological samples with associate advantages, it is still a matter of debate, if all EUS guided punctures should be done with these (costly) second generation FNB needles because of their superior diagnostic potential. A meta-analysis by Khan et al found, that there was no difference in diagnostic yield between FNA and FNB, but only when FNA is accompanied by rapid onsite evaluation (ROSE).^[7]^ In their publication, one of the most significant studies concerning diagnostic accuracy of EUS-FNA with incorporation of an onsite cytopathologist showed a diagnostic accuracy of 93,8%.^[24]^ Interestingly, this study was an outlier in terms of EUS-FNA performance and therefore not included in the meta-analysis by Khan et al.^[7]^ In our study using FNA needles with LBC, we achieved comparably high performance in terms of diagnostic accuracy of 92,8% without involving an onsite cytopathologist. Therefore, we hypothesize tissue processing using LBC may bypass the presence of a cytopathologist and may achieve comparable diagnostic performance in comparison to both FNA/ROSE and FNB. This hypothesis is supported by a recently published study of Tomita et al showing that the diagnostic performance of FNA when combined with LBC is comparable to FNB.^[25]^ In line, Arena et al found no additional benefit of an on-site cytopathologist in terms of accuracy when FNB is compared to FNB with ROSE.^[26]^

While FNB needles enable the acquisition of larger specimens on which to perform immunohistochemical and molecular analyses,^[6]^ LBC likewise allows to perform these advanced staining protocols for a detailed characterization of cytospin pellets.^[8]^

Our study has several strengths and limitations. The large consecutive cohort of 172 patients is one of the major strengths of our study. Moreover, our cohort represents a typical distribution of benign and malignant pancreatic diseases presented in daily clinical practice. Also, there was no change in the high endoscopists level of experience since all remained the same over the entire study period. Furthermore, technical aspects of tissue acquiring and used equipment (needles etc) did not change either. A major limitation is the retrospective design of the study. Moreover, since LBC specimen were prepared by experienced pathologists while direct smear of specimen on glass object plates was performed by the endoscopist himself, this additional difference in tissue processing might have influenced study results, even if representing a typical workflow in daily clinical care.

## 5. Conclusion

Liquid based cytology is a simple technique that helps improving sensitivity, specificity, NPV and accuracy over conventional cytology in fine needle aspirates from patients with pancreatic lesions. Also, it may bypass the need for rapid onsite evaluation of specimen performed by an onsite cytopathologist and may be equivalent to the use of FNB. Further multicentric investigations, especially comparing the performance of FNA combined with LBC to ROSE or FNB, resp., are necessary.

## Author contributions

Dr Martin Bürger:

-Conception and design

-Acquisition of data

-Analysis of data

-Endoscopic investigations

-Interpretation of data

-Drafting the work

-Final approval of the manuscript

Dr Antje Heidrich:

-Acquisition of data

-Interpretation of data

-Revising the work

-Final approval of the manuscript

Prof Dr Andreas Stallmach:

-Acquisition of data

-Endoscopic investigations

-Interpretation of data

-Revising the work

-Final approval of the manuscript

Prof Dr Iver Petersen:

-Analysis of specimen

-Interpretation of data

-Revising the work

-Final approval of the manuscript

PD Dr Carsten Schmidt:

-Conception and design

-Acquisition of data

-Endoscopic investigations

-Analysis of data

-Interpretation of data

-Drafting the work

-Final approval of the manuscript

-Statistical analysis
